# A T-Cell Surface Marker Panel Predicts Murine Acute Graft-Versus-Host Disease

**DOI:** 10.3389/fimmu.2020.593321

**Published:** 2021-01-29

**Authors:** Carina A. Bäuerlein, Musga Qureischi, Zeinab Mokhtari, Paula Tabares, Christian Brede, Ana-Laura Jordán Garrote, Simone S. Riedel, Martin Chopra, Simone Reu, Anja Mottok, Estibaliz Arellano-Viera, Carolin Graf, Miriam Kurzwart, Katharina Schmiedgen, Hermann Einsele, Matthias Wölfl, Paul-Gerhardt Schlegel, Andreas Beilhack

**Affiliations:** ^1^ Department of Medicine II, University Hospital of Würzburg, Würzburg, Germany; ^2^ Interdisciplinary Center for Clinical Research (IZKF), Würzburg University, Würzburg, Germany; ^3^ Graduate School of Life Sciences, Würzburg University, Würzburg, Germany; ^4^ Institute of Pathology, Würzburg University, Würzburg, Germany; ^5^ Department of Pediatrics, University Hospital of Würzburg, Würzburg, Germany

**Keywords:** acute graft-versus-host disease, alloreactive T cells, transplantation, prediction, mouse models

## Abstract

Acute graft-versus-host disease (aGvHD) is a severe and often life-threatening complication of allogeneic hematopoietic cell transplantation (allo-HCT). AGvHD is mediated by alloreactive donor T-cells targeting predominantly the gastrointestinal tract, liver, and skin. Recent work in mice and patients undergoing allo-HCT showed that alloreactive T-cells can be identified by the expression of α4β7 integrin on T-cells even before manifestation of an aGvHD. Here, we investigated whether the detection of a combination of the expression of T-cell surface markers on peripheral blood (PB) CD8^+^ T-cells would improve the ability to predict aGvHD. To this end, we employed two independent preclinical models of minor histocompatibility antigen mismatched allo-HCT following myeloablative conditioning. Expression profiles of integrins, selectins, chemokine receptors, and activation markers of PB donor T-cells were measured with multiparameter flow cytometry at multiple time points before the onset of clinical aGvHD symptoms. In both allo-HCT models, we demonstrated a significant upregulation of α4β7 integrin, CD162E, CD162P, and conversely, a downregulation of CD62L on donor T-cells, which could be correlated with the development of aGvHD. Other surface markers, such as CD25, CD69, and CC-chemokine receptors were not found to be predictive markers. Based on these preclinical data from mouse models, we propose a surface marker panel on peripheral blood T-cells after allo-HCT combining α4β7 integrin with CD62L, CD162E, and CD162P (cutaneous lymphocyte antigens, CLA, in humans) to identify patients at risk for developing aGvHD early after allo-HCT.

## Introduction

Allogeneic hematopoietic cell transplantation (allo-HCT) has proven as a curative therapy for life-threatening diseases of the hematopoietic system such as leukemia and lymphomas (1, 2). However, acute graft-*versus*-host disease (aGvHD) remains the leading cause of death of transplant-related complications resulting in up to 20% mortality after allo-HCT (3, 4). As early prediction of aGvHD remains difficult, potent preemptive immunosuppressive regimens have been established that require swift adaptation as soon as first clinical aGvHD symptoms appear. Intensifying immunosuppression early, however, puts patients at high risk of infectious complications, whereas late treatment often comes too late to avoid disease exacerbation. Considering the occurrence of aGvHD as an end-result of a forceful and highly dynamic immune response may explain how difficult it is to treat patients once aGvHD occurs. Preclinical studies in animal models have revealed a pathophysiological cascade of events that precede aGvHD onset. First, host conditioning triggers responses to tissue injury such as neoangiogenesis (5), recruitment of innate immune cells, such as neutrophilic granulocytes and monocytes (6–8), and release of cytokines and cytokine receptors (9). Concomitantly, naïve donor T-cells migrate to secondary lymphoid organs (SLOs), undergo allo-antigen priming, proliferate, differentiate into effector T-cells, and migrate from these priming sites to peripheral tissues such as the intestinal tract, liver, and skin, where they cause organ damage resulting in clinical aGvHD manifestation (10–13).

Thus, to improve clinical outcome it had become clear that identifying and monitoring predictive markers to identify patients at risk of developing clinically relevant aGvHD are warranted and these endeavors are starting to bear fruits (14–16). Tissue damage, release of damage-associated factors and cytokines can be picked up by proteomic and systems biology analysis approaches to guide treatment decisions and provide a basis for specific therapeutic interventions (17).

Recent insights into aGvHD pathophysiology supported by observations in mouse models uncovered a temporally and spatially defined aGvHD initiation and effector phase (11, 13, 18). Extensive studies of the initiation and effector phases of alloreactive T-cells have revealed that after priming of alloreactive T-cells in SLOs, donor T-cells are mobilized into the peripheral blood (PB) before homing to target tissues of aGvHD. In minor histocompatibility antigen mismatch (miHAg) allo-HCT mouse models the transition from initiation of aGvHD to the aGvHD effector phase can last for an extended period of two weeks before the first clinical symptoms of aGvHD occur (19). In this transition phase alloreactive T-cells are circulating in the PB before entering target organs. This phenomenon can also be observed in patients before the onset of clinically apparent aGvHD (20). The timely homing of alloreactive donor T-cells to aGvHD target tissues depends on the combination of the appropriate surface receptor expression profile (21). We hypothesized that these homing receptors could provide a unique footprint to distinguish alloreactive T-cells in the PB. As distinct surface receptors have been functionally implicated in the development of aGvHD, the close monitoring of PB T-cells and their unique surface marker profile might be an appropriate approach to predict the risk of aGvHD even before the release of tissue damage associated surface markers. Indeed, Paszesny and colleagues identified a CD4^+^CD146^+^CCR5^+^ T-cell population in the peripheral blood before the onset of aGvHD that predicted patient outcome (22).

Here, we investigated surface receptors on PB CD8^+^ T-cells in two independent mouse models after miHAg allo-HCT before aGvHD symptoms appear. We identified a combination of homing receptors, namely α4β7 integrin, CD162E, CD162P, and CD62L, on PB CD8^+^ T-cells that may serve as an early potential biomarker panel to predict onset of aGvHD. Surprisingly, CC-chemokine receptors deemed not suitable to identify alloreactive CD8^+^ T-cells. Our observations provide the basis to prospectively evaluate corresponding surface receptors on T-cells in patients undergoing allo-HCT to unambiguously identify a surge of alloreactive T-cells before clinical symptoms occur.

## Materials and Methods

### Mice

BALB/b (H-2^b^, CD90.2), B10.D2 (H-2^d^, CD90.2), and congenic C57Bl/6 (B6, H-2^b^, CD90.1) mice were obtained from Jackson Laboratories (Bar Harbor, ME), C57Bl/6 (B6, H-2^b^, CD90.2), and BALB/c (H-2^d^, CD90.2) mice were obtained from Charles River (Sulzfeld, Germany). All mice were housed in a pathogen-free facility at the Center for Experimental Molecular Medicine (ZEMM), Würzburg. All experiments were performed according to the German regulations for animal experimentation and governmental approval (Permit 55.2-2531.01-30/09, 55.2-2531.01-82/14, and 55.2.2-2532-2-537-22).

### Allogeneic and Syngeneic HCT

8- to 12-week-old female recipient mice were myeloablatively irradiated (BALB/b, BALB/c, B10.D2 8 Gy; B6 9 Gy) with an electron linear accelerator (Mevatron Primus, Siemens, Germany) prior to HCT. For hematopoietic reconstitution B6→BALB/b allo-HCT recipients (or syngeneic B6 controls) were injected intravenously with 5 x 10^6^ WT B6 bone marrow (BM) cells and 5 x 10^6^ splenic CD4^+^ and CD8^+^ B6 T-cells (expressing the congeneic marker CD90.1). Myeloablatively conditioned B10.D2→BALB/c allo-HCT recipients (or syngeneic B10.D2 controls) were transplanted with 5 x 10^6^ BM cells and 3 x 10^7^ splenocytes (containing an equivalent of 1 x 10^7^ CD4^+^ and CD8^+^ donor T-cells without congeneic marker). Bone marrow controls received 5 x 10^6^ BM cells only. Transplanted mice were monitored daily for survival, weight change, and clinical aGvHD symptoms according to Cooke et al (23).

### Histopathological Analysis

Mice were euthanized on day +25 after HCT and organs were fixed in 4% PFA (Paraformaldehyde) in PBS. Tissues were cut into 2 µm sections and stained with H&E (hematoxylin and eosin). GvHD scoring was performed according to Lerner et al. (24). Healthy tissue was considered as 0, whereas mild and moderate tissue damage were graded with 1–2. Severe tissue damage was considered as 3. All samples were graded by unbiased pathologists in a blinded fashion.

### Flow Cytometry

Peripheral blood samples of murine HCT recipients and untreated controls were collected at indicated time points. Erythrocytes were lysed and after washing cells were blocked with normal rat serum and stained with appropriate antibodies at 4°C for 30min. The following anti-mouse antibodies (clones) were used: anti-CD3ϵ (17A2), anti-CD8α (53-6.7), anti-CD4 (RM4-5), anti-CD45.1 (A20), anti-LPAM-1/α4β7 (DATK32), anti-CD103/αEβ7 (2E7), anti-CCR4 (2G12), anti-CCR5 (C34-3448), anti-CCR7 (4B12), anti-CD25 (PC61), anti-CD44 (IM7), anti-CD62L (MEL-14), anti-CD69 (H1.2F3) (Biolegend, Uithoorn, Netherlands); anti-CD90.1 (H1S51) (eBioscience, Frankfurt, Germany); anti-CCR2 (475301), anti-CCR6 (FAB590P), anti-CCR9 (242503), E-selectin ligand-Fc chimera (R&D Systems, Germany), P-selectin ligand-IgG fusion protein (BD, Heidelberg, Germany); anti-human-IgG-FITC (Jackson ImmunoResearch Laboratories, West Grove, PA). Dead cells were excluded with propidium iodide (PI, Invitrogen, Darmstadt, Germany) or Zombie Aqua™ Fixable Viability Kit staining (Biolegend). Flow cytometry was performed on a FACS Canto II (BD), and data was analyzed with FlowJo (BD) and Infinicyt Software (Cytognos, Salamanca, Spain). Gates were set using the fluorescence-minus-one gating strategy. Anti-mouse or anti-rat/hamster CompBeads (BD) were used for compensation.

### Statistical Analyses, Principal Component Analysis and K-Means Data Clustering

Repeated measurements are expressed as the mean ± standard deviation. To compare miHAg mismatch versus syngeneic recipients for several time points, a repeated measures ANOVA was conducted with a posthoc analysis using a Bonferroni correction of the significance level. Data was analyzed with GraphPad Prism 9 software (La Jolla, CA, USA) and IBM SPSS 22 (Armonk, NY, USA). Data reaching statistical significance is indicated as *P ≤ 0.05, **P ≤ 0.01, ***P ≤ 0.001.

To extract the variance of a data set, multivariate principal component analysis (PCA) was employed. Data dimensionality was reduced while retaining the original data information. Each sample was displayed by a data point in a 2D principal component space (PC1 and PC2) and data points with similar characteristics were grouped together while the different data groups were visualized in PC space.

Unsupervised K-means clustering was employed to partition the unlabeled data set into K clusters where the mean distances between data points in the same cluster was minimized.

## Results

### Onset of aGvHD in Two miHAg Mismatch Allo-HCT Mouse Models

**Figure 1:**
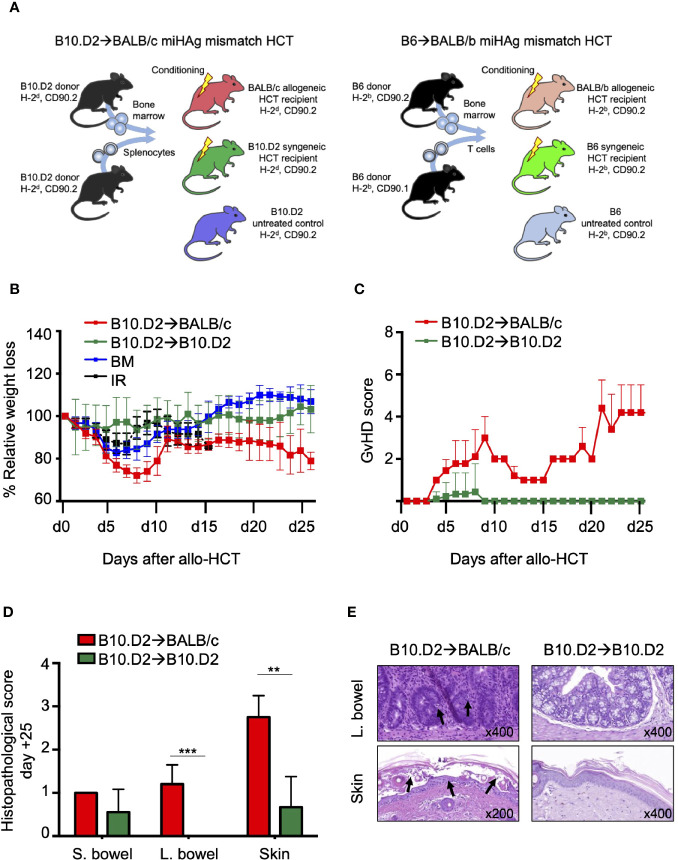
Murine MHC matched miHAg mismatched allo-HCT models to assess a biomarker panel on peripheral blood (PB) CD8^+^ T-cells for aGvHD. **(A)** Schematic overview of the two described miHAg mismatch allo-HCT and syngeneic HCT models. **(B–E)** Validation of aGvHD induction in the B10.D2→BALB/c miHAg mismatch allo-HCT model. **(B)** Weight loss relative to day 0 of HCT of the B10.D2→BALB/c model and the respective control groups. **(C)** Clinical scores during aGvHD progression of miHAg mismatch and syngeneic recipients. **(D)** Histopathological grading of the aGvHD target organs intestine and skin on day +25 after allo-HCT and **(E)** representative hematoxylin-eosin stains at indicated magnification. Values are displayed as means ± SD. MiHAg mismatch B10.D2→B10.D2 BM+T-cells, n = 9; syngeneic B10.D2→B10.D2 BM+T-cells, n = 9; BM only, n = 5; irradiation only, n = 5. T = T-cells, BM = Bone Marrow, IR = Irradiation.

GvHD target organ infiltration depends on the appropriate expression of homing receptors on migrating cells (21). Therefore, we tested an extended panel of surface markers consisting of adhesion molecules, chemokine receptors, and activation markers to determine potential biomarkers that may specifically define alloreactive T-cells in the PB. To this end, we compared two CD4^+^ and CD8^+^ T-cell dependent miHAg mismatched allogeneic mouse models (B6→BALB/b and B10.D2→BALB/c, **Figure 1A**) with syngeneic HCT recipients and healthy WT controls. Based on our previous results of donor T-cell migration kinetics (19), we chose two peak time points during the early and later phase of T-cell mobilization to the PB (days+6 and +15) and included day +21 (clinically apparent aGvHD onset after murine miHAg allo-HCT) as appropriate for a precise receptor analysis.

First, we analyzed whether miHAg B10.D2→BALB/c allo-HCT showed the same aGvHD kinetics as the previously reported B6→BALB/b model (19). Host conditioning regimen and transplantation procedure resulted in 20%–30% weight loss during the first 8 days after allo-HCT in B10.D2→BALB/c miHAg mismatched recipients of BM and T-cells. Mice recovered after 10 days and reached 80%–90% of their initial body weight but could not recover completely. Weight remained at the same level until the end of the experiment. In contrast, weight loss of syngeneic B10.D2→B10.D2 recipients was rather mild during the first week after HCT (10%) compared to BM recipients (15%–20%). Both groups recovered 10 days after HCT and reached their starting weight (**Figure 1B**). In allo-HCT recipients, typical signs of aGvHD occurred around day+20. Symptoms included diarrhea, skin inflammation, and ruffled fur (**Figure 1C**).

Histopathological analysis confirmed aGvHD in the small and large bowel as well as the skin on day+25 after B10.D2→BALB/c allo-HCT (**Figures 1D, E** **)**. Increased tissue damage, T-cell infiltration, and crypt apoptosis contributed to the significant aGvHD histological score in the large bowel whereas typical signs of aGvHD in the skin featured necrotic keratinocytes, inflammation, and basal vacuolization of the epidermis.

### Alloreactive Donor CD8^+^ T-Cells Upregulate Homing Molecules Early After HCT

**Figure 2:**
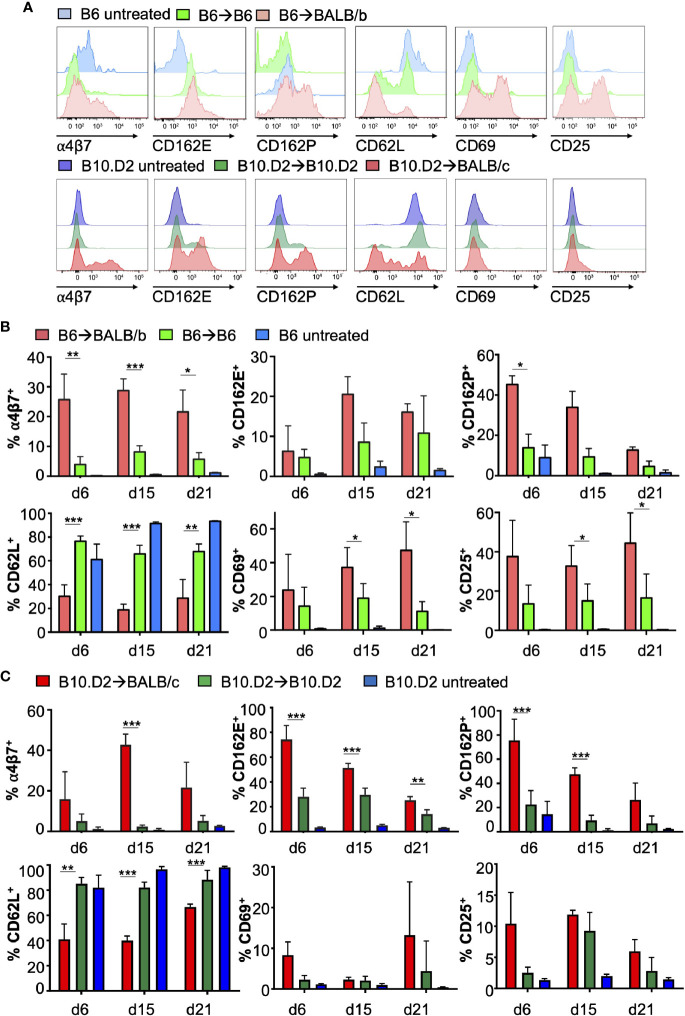
Allogeneic peripheral blood CD8^+^ T-cells display a distinct surface marker profile before the onset of aGvHD. **(A)** Representative histograms show surface receptor expression on PB CD8^+^ T-cells on day +15 (upper row B6 → BALB/B model, lower row B10.D2→BALB/c model) after allo-HCT compared to the respective expression levels of syngeneic and untreated healthy controls. **(B, C)** Summary of surface receptor expression on PB CD8^+^ T-cells at indicated time points after HCT and untreated controls. Values are displayed as means ± SD. C57Bl/6→BALB/b: n= 3–6 in each group, B10.D2→BALB/c: n = 5–9 in miHAg mismatch and syngeneic control group, untreated B10.D2 n = 4. Statistical significance between miHAg mismatch recipients and syngeneic controls was determined with a repeated ANOVA and posthoc analysis using Bonferroni correction of the significance level. *P ≤ 0.05; ** *P* ≤ 0.01; *** *P* ≤ 0.001.

Next, we collected blood samples of the tail vein from allogeneic and syngeneic recipients on days+6, +15, and +21 for immune phenotyping of PB CD8^+^ T-cells (gating strategy for the B6→BALB/b model is shown in **Supplementary Figure 1**) and compared these to the respective untreated controls (**Figure 2**).

PB CD8^+^ T-cells highly up-regulated the mucosal homing receptor α4β7 integrin after miHAg mismatch allo-HCT (**Figure 2A**, representative histograms) in both miHAg models. In the allogeneic B6→BALB/b model, α4β7^+^ CD8^+^ T-cell frequencies in the PB significantly exceeded those after syngeneic HCT at all time points and the expression peaked at day+15 (28.7 ± 3.9% vs. 8.1 ± 2.1%, *P* < 0.0001; < 1% α4β7^+^PB CD8^+^ T-cells in untreated controls, **Figure 2B**, **Supplementary Figure 2**). Similarly, PB CD8^+^ T-cells of B10.D2→BALB/c miHAg mismatch recipients expressed significantly higher levels of α4β7 integrin compared to syngeneic controls at all measured time points and α4β7 expression peaked on day+15 (42.72 ± 5.3% vs. 2.4 ± 0.8%, *P* = 0.0002, **Figure 2C**). Next, we analyzed expression levels of the skin homing receptors CD162E and CD162P after allo-HCT. Notably, in B10.D2→BALB/c miHAg allo-HCT recipients, CD162E expression levels of alloreactive PB CD8^+^ T-cells exceeded significantly the levels found in syngeneic controls at all measured time points (e.g., 74.3 ± 11.2% vs. 28.1 ± 6.95% on day+6, *P* < 0.0001) (**Figures 2A, C** **)**. On day+6, in B6→BALB/b miHAg allo-HCT recipients, CD162E^+^ CD8^+^ T-cell frequencies did not differ between allogeneic and syngeneic HCT recipients (**Figure 2B**). However, thereafter, levels of CD162E^+^ T-cells after miHAg mismatch allo-HCT exceeded those found in syngeneic controls (20.5 ± 4.4% versus 8.5 ± 4.7% on day+15, p = 0.1). By day+21, CD162E expression levels almost equaled between both groups again. In contrast, in untreated controls less than 5% of PB CD8^+^ T-cells expressed CD162E under steady-state conditions.

Additionally, PB CD8^+^ T-cells highly up-regulated CD162P in both miHAg allo-HCT models (**Figures 2B, C** **)** compared to syngeneic controls. In B6→BALB/b miHAg allo-HCT recipients, 45.2 ± 4.4% of CD8^+^ T-cells expressed CD162P already on day+6 compared to 13.8 ± 6.8% of CD8^+^ T-cells in syngeneic controls (*P* = 0.01) and less than 10% of CD8^+^ T-cells in untreated controls. Already by day+6 in B10.D2→BALB/c allo-HCT, CD8^+^CD162P^+^ expression levels of allo-HCT recipients significantly exceeded those of syngeneic recipients (75.6 ± 17.4% vs 22.6 ± 11.4%; *P* = 0.0001).

Furthermore, the lymphoid homing receptor CD62L significantly decreased on PB CD8^+^ T-cells after allo-HCT and remained significantly lower compared to syngeneic HCT at all analyzed time points (30.2 ± 9.6% vs. 76.4 ± 4.4%, *P* = < 0.0001 on day+6 after B6→BALB/b allo-HCT and 41 ± 12.3% vs. 85.2 ± 5%, *P* = 0.0019 on day+6 after B10.D2→BALB/c allo-HCT, respectively). In syngeneic recipients, PB CD8^+^CD62L^+^ T-cell frequencies were only slightly lower than those of untreated controls (75 to 95% CD62L^+^).

To assess the activation status of PB CD8^+^ T-cells we investigated the expression of CD69 and CD25. Frequencies of PB CD8^+^CD69^+^ T-cells differed significantly between the two groups only on day+15 (37.1 ± 11.8% in allogeneic B6→BALB/b vs. 18.9 ± 8.7% in syngeneic recipients, *P* = 0.0409; < 3% of circulating CD8^+^ CD69^+^ T-cells in untreated controls) and +21 (**Figure 2B**). After B10.D2→BALB/c allo-HCT, CD69 expression exceeded the levels measured on PB CD8^+^ T-cells of syngeneic controls on day+6 (8.3 ± 3.2 vs. 2.3 ± 1.1; *P* = 0.08, representative histograms in **Figure 2A**).

PB CD8^+^ T-cells of B6→BALB/b allo-HCT recipients significantly up-regulated the activation marker CD25 compared to syngeneic controls on days+15 and day +21 (32.7 ± 10.5% CD8^+^CD25^+^ T-cells in allogeneic vs. 15.03 ± 8.7% in syngeneic recipients, *P* = 0.03, **Figure 2B**). Yet, no significant differences between allogeneic and syngeneic recipients were determined after B10.D2→BALB/c allo-HCT (**Figure 2C**).

Other markers such as CC-chemokine receptors (CCRs 2, 4, 5, 6, 7, 9) surprisingly never differed between allogeneic and syngeneic recipients (**Supplementary Figure 3**). At day+6, PB CD8^+^ T-cells highly up-regulated all CCRs both in allogeneic B6→BALB/b recipients and in syngeneic B6→B6 controls compared to untreated animals. After day+6, CCR expression levels decreased similarly in allogeneic and syngeneic recipients. The differences in CCR expression levels did not reach statistical significance at any of the analyzed time points.

### Combined Surface Marker Panel Predicts aGvHD Onset

**Figure 3:**
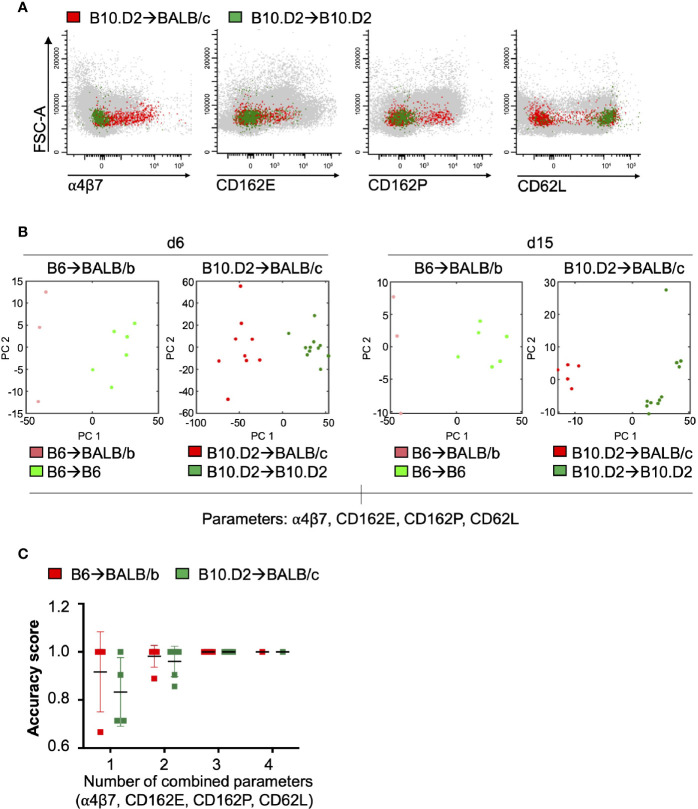
Combination of surface receptors defines alloreactive T-cells in the PB of allo-HCT recipient. **(A)** Representative FACS plots of PB samples on day+15 analyzed with Infinicyte Flow Cytometry Software. Overlay of allogeneic (red) vs. syngeneic (green) CD3^+^CD8^+^ T-cells of total peripheral blood mononuclear cells (PBMC, in grey). **(B)** Principal component analysis based on four parameters and unsupervised K-means clustering of individual samples. Each data point represents one mouse. Red dots show allogeneic and green dots show syngeneic recipients of both models. Distances between data points represent the similarity. **(C)** Determination of accuracy score [= (number of true classified samples)/(number of total test data)] for the combination of parameters. Each point represents one possible marker combination.

Based on these results, we next asked whether the combination of T-cell surface markers can clearly discriminate allogeneic transplanted mice from syngeneic recipients and untreated controls (**Figure 3A**). Our principle component analysis considered the four surface markers α4β7 integrin, CD162E, CD162P, and CD62L as CD25, CD69, and CC-chemokine receptors were not exclusively expressed on alloreactive T-cells. To classify the animals based on their treatment, we applied an unsupervised K-means clustering on the percentage of CD8^+^ T-cells with high expression levels of the four aforementioned surface markers for day+6 as an early GvHD prediction would be most beneficial. We compared the clustering based on four markers to a clustering based on the single marker α4β7 integrin to investigate if the combination of several markers could enhance the predictive value (**Figure 3B**). The first two principal component spaces (PC1 and PC2) predict the variance in the data. The clustering analysis based on α4β7 integrin alone could unequivocally separate allogeneic from syngeneic recipients on day+15 but not on day+6. Determining the accuracy score [= (number of true classified samples)/ (number of total test data)] for several marker combinations on day+6 showed that in both models the maximum prediction accuracy could be obtained with a minimum of three parameters (**Figure 3C**). Conclusively, our data suggest that the early upregulation of α4β7 integrin, CD162E, CD162P as well as low expression levels of CD62L on circulating PB CD8^+^ T-cells serve as strong predictor of aGvHD and that the combination of at least three markers increases the precision of our analysis.

## Discussion

**Figure 4:**
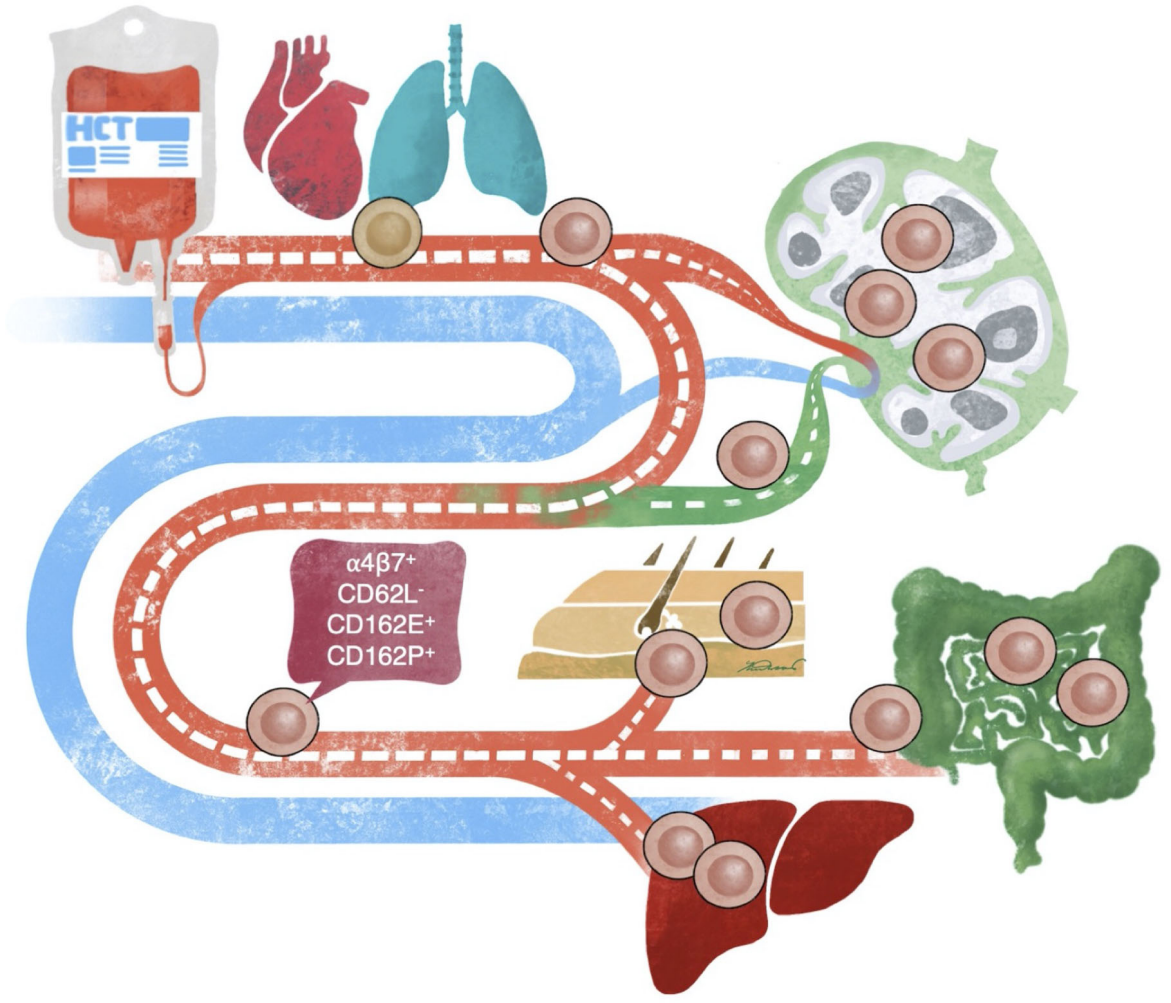
Proposed biomarkers to measure circulating alloreactive CD8^+^ T-cells before aGvHD onset. Based on our results we propose a panel of α4β7 integrin, CD62L, CD162E, and CD162P CD8^+^ T-cell surface markers to predict onset of aGvHD as early as day +6 after allo-HCT. The proposed marker panel relies on data from two independent CD4^+^ and CD8^+^ T-cell driven miHAg allo-HCT mouse models. Notably, T-cell reconstitution after allo-HCT can take four weeks or even longer. However, upon antigen contact T-cell mediated alloimmune responses take only a few days to be unleashed allowing for the discrimination of alloreactive vs. reconstituting T-cell populations. For translation of our findings into clinical studies it will be important to factor in the heterogeneity of the patient population in terms of patient history, conditioning regimens, genetics, and immunosuppressive GvHD prophylaxis.

In this study, we present a combinatorial panel of α4β7 integrin, CD162E, CD162P, and CD62L as predictive to unequivocally identify alloreactive CD8^+^ T-cells in the PB of murine allo-HCT recipients (**Figure 4**). Benefiting from insights into donor T-cell migration kinetics in the PB early after HCT (19), we analyzed the dynamic changes of surface markers on donor CD8^+^ T-cells after miHAg mismatch allo-HCT. The combination of these markers proved superior to single markers especially at early time points after allo-HCT allowing a reliable prediction for early time points. Examining if the combination of the expression of chemokine receptors, homing and activation markers could give more defined insights into T-cell homing to specific target tissues of aGvHD we confirmed in two independent miHAg allo-HCT models that the expression of α4β7 integrin, CD162E, CD162P, and lack of CD62L on PB CD8^+^ T-cells preceded the onset of aGvHD. Conversely, we could exclude an extended panel of surface receptors (several chemokine receptors, CD69, and CD25) as these did not turn out as reliable predictors.

### Are These Identified Markers Functionally Linked to aGvHD Pathophysiology?

Our analysis in two independent murine models of aGvHD, confirmed previous studies in mice showing that alloreactive CD8^+^ donor T-cells highly up-regulate α4β7 integrin at early time points after HCT (25–27). In patients, upregulation of α4β7 integrin on conventional T-cells or reduction of α4β7^+^ Tregs, respectively, correlated with intestinal aGvHD manifestation (28–30). Accordingly, α4β7 integrin also proved as a relevant therapeutic target for the treatment of intestinal inflammation. Vedolizumab, a monoclonal antibody against α4β7, is approved for ulcerative colitis and under current investigation for the preemptive therapy of aGvHD (NCT03657160, ClinicalTrials.gov). The frequency of α4β7^+^ T-cells in the PB of colitis ulcerosa patients before Vedolizumab treatment could predict treatment outcome (31). Therefore, α4β7 integrin not only appears as an attractive diagnostic and prognostic biomarker for intestinal aGvHD but may also be predictive for treatment outcome.

In both murine miHAg allo-HCT models, allogeneic circulating PB CD8^+^ T-cells upregulated the skin homing receptors CD162E and CD162P. Both mouse surface molecules (CD162E, CD162P) expressed by allogeneic donor T-cells or the corresponding human skin homing receptor CLA, respectively, are strongly associated with aGvHD initiation (32, 33), even if redundancies with other homing pathways have been observed (34). Patients suffering from aGvHD showed higher levels of circulating CLA^+^ T-cells compared to patients who did not develop aGvHD (35).

The lymphoid homing receptor CD62L is expressed on naïve, central memory T-cells but also on T-cells with a stem-cell like memory phenotype (36). Enhanced mobilization of CD8^+^CD44^hi^CD62L^low^ effector T-cells and a reduced number of even circulating CD62L^+^ naïve T-cells were demonstrated to be a strong indicator of aGvHD days and in our hands even two weeks before the onset of aGvHD.

However, as a cautionary note in the interpretation of our presented data, we have to point out that in both independent miHAg allo-HCT GvHD models, mice developed intestinal, liver and skin aGvHD. Therefore, the proposed panel may have prognostic value that there is an increased risk of aGvHD development, but at this stage of our study it is too early to draw conclusions about the specific organ manifestation.

### Are More T-Cell Biomarkers Better Than One?

In our unbiased clustering analysis, α4β7 integrin alone could not robustly separate allogeneic from syngeneic recipients on day+6 after allo-HCT two weeks before clinical aGvHD onset, whereas the combination of surface markers proved highly reliable [α4β7, CD162E/CD162P (CLA) and CD62L]. By determination of the accuracy parameter we observed the maximum precision of our predictive test when we combined three markers. However, we have observed already a high accuracy score (0.87 ± 0.15) for only one single parameter, which might be explained by our well-controlled experimental setting and the relatively low sample size. Increasing the sample size and keeping in mind the heterogeneity of patient samples, we propose to increase the number of measured parameters to a minimum of three parameters.

As the accuracy score for three combined markers reached already the maximum and did not change by adding another marker in both models, it is tempting to limit future testing to a panel of three surface markers on PB CD8^+^ T cells. However at this stage, as pointed out above, the panel cannot predict site-specific aGvHD manifestation yet. As in the clinical scenarios, one can expect a much higher heterogeneity in patient samples, we would recommend to consider more than three markers for an exploratory patient study, even if a stringent consideration of three markers may turn out sufficient as in our preclinical mouse models to predict aGvHD onset. In summary, based on our results, we expect that a combined marker panel of at least three markers will prove valuable for an early prediction of aGvHD in allograft recipients.

### What Are the Limitations of This Study?

First, while our results were highly reproducible in two independent mouse models, the situation in human patients undergoing allo-HCT is certainly more complex (37). Both miHAg allo-HCT mouse models and syngeneic controls relied on a myeloablative irradiation only-based conditioning regimen, whereas in patients a variety of conditioning protocols are used. Second, genetic uniformity of donors and recipients utilizing inbred mouse strains contrast to the enormous genetic heterogeneity in patients who also vary in their clinical history, age, underlying diseases, co-morbidities, and previous medications. Third, mice were housed in homogenous specific pathogen-free microbial conditions and did not receive any immunosuppressive GvHD prophylaxis. Steroids can have an impact on the expression levels of homing receptors (38). Furthermore, by design of our study, mice did not suffer from malignant diseases or succumbed to infections. Future studies will have to address whether the panel of T cell markers allows discrimination between allo-HCT patients at risk for aGvHD and patients suffering from opportunistic infections or both, e.g., increased risk for aGvHD development and/or cytomegalovirus (CMV)-reactivation. Biomarkers for the beneficial graft-versus-leukemia response and the efficient combat of opportunistic infections still need to be defined. However, a reductionist approach employing miHAg GvHD mouse models has the advantage to evaluate the reproducibility of candidate biomarkers. By utilizing one miHAg allo-HCT model for a discovery set of biomarkers and the second miHAg allo-HCT model for validation, these models helped to identify a panel of biomarkers that now need to be rigorously tested in patients while ruling out a range of preconceived candidates.

In contrast to the analyzed integrin and selectin family members, none of the tested CC-chemokine receptors could give more insights into T-cell homing to different tissues or GvHD onset. This may be partially explained by their dynamic regulation, vulnerability to loss of expression and artifacts upon *ex vivo* cell manipulation and redundant expression of CCRs (39). Yet, inflammation after conditioning appears to trigger CCR and endothelial ligand expression (40, 41), consistent with our observed CCR up-regulation in both allogeneic and syngeneic recipients. Of note, over time CCR expression decreased, suggesting that host conditioning upregulated CCRs on CD8 T-cells independent of the allogeneic or syngeneic setting. Nevertheless, CCRs still hold the potential as therapeutic targets. For instance, Reshef et al. reported the beneficial effects of CCR5 blockade, which resulted in reduced aGvHD in phase I/II clinical trials (42, 43). These effects were consistent with previously published results in murine models (44, 45).

### How Can These Findings Be Translated to the Clinics?

Unbiased clustering analysis revealed a panel of four CD8^+^ T-cell surface markers (α4β7, CD162E/CD162P (CLA), CD62L) in two independent miHAg allo-HCT models. This suggests that flow cytometric analysis of PB T-cells also in patients undergoing allo-HCT could predict aGvHD before patients become symptomatic. Based on these results, we recommend to prospectively test the combination of the aforementioned surface molecules in allo-HCT patients at early time points. Cytotoxic CD8^+^ effector T-cells are key players in aGvHD pathophysiology. Thus, it appears attractive to design GvHD prophylaxis protocols based on the detection of circulating alloreactive T-cells, even before they can exert their tissue destructive force. As time to onset of aGvHD markedly varies between patients, it will be important to design a prospective clinical trial based on a calendar-driven collection to standardize data acquisition plus an event-driven sample collection. Second, as efficient homing of immune effector cells is a prerequisite to combat infections and to exert the desired GvL effect, a vigorous prospective study will require a relatively large sample size and meticulous clinical and laboratory documentation to discern aGvHD causing alloimmune responses from these scenarios. Furthermore, it will be interesting to compare and cross-validate the proposed panel of four surface flow cytometric biomarkers with currently investigated serum and urine biomarkers (46–48). Assessment of T-cell surface markers in real-time will help to bring down GvHD-related mortality by early recognition and intervention.

## Data Availability Statement

The original contributions presented in the study are included in the article/**Supplementary Material**. Further inquiries can be directed to the corresponding author.

## Ethics Statement

All experiments were performed according to the German regulations for animal experimentation and governmental approval (Permit 55.2-2531.01-30/09, 55.2-2531.01-82/14 and 55.2.2-2532-2-537-22).

## Author Contributions

CB and MQ performed and CB, A-LJG, SSR, MC, EA-V, CG, MK, KS, and MW helped with experiments. SR and AM performed histopathologic analyses. CB, MQ, ZM, MW, and AB analyzed data, designed research, and wrote the paper. P-GS and HE helped write the paper. All authors contributed to the article and approved the submitted version.

## Funding

This study was supported by a Deutsche José Carreras Leukämie-Stiftung (DJCLS F 11/04) research fellowship to CB, grants of the Wilhelm-Sander-Stiftung (2007–081), Interdisciplinary Center for Clinical Research Würzburg (IZKF, B-233) and the Deutsche Forschungsgemeinschaft DFG SFB TR221 (Project number 324392634: B09, Z02 and A03), the Europäische Fonds für Regionale Entwicklung (EFRE) and an unrestricted donation by Merete and Alexander Knauf to AB. MQ was awarded with a PhD fellowship from the Graduate School of Life Sciences Würzburg of the German Excellence Initiative.

## Conflict of Interest

The authors declare that the research was conducted in the absence of any commercial or financial relationships that could be construed as a potential conflict of interest.
