# Supplementary material for: A T-Cell Surface Marker Panel Predicts Murine Acute Graft-Versus-Host Disease
Source: Front Immunol. 2021 Jan 29;11:593321. doi: 10.3389/fimmu.2020.593321 (PMC7880247; doi:10.3389/fimmu.2020.593321)
Supplement: Supplementary file 1 [file DataSheet_1.pdf]

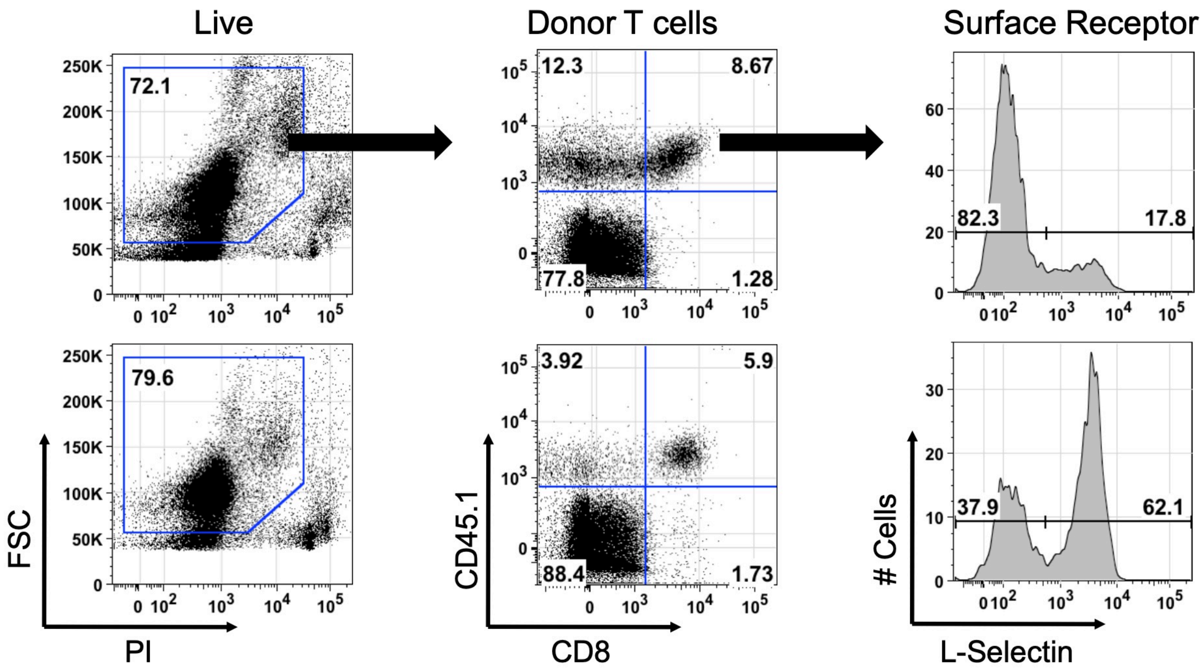


**Supplementary Figure 1: Gating strategy to distinguish donor from host CD8$^+$ T cells after B6→BALB/b allo-HCT vs. B6→B6 syngeneic HCT.**

Representative FACS plots show the gating strategy for one allogeneic (upper row) and one syngeneic (lower row) recipient. Live CD8$^+$ donor T-cells (PI$^-$, CD45.1$^+$, CD8$^+$) in the PB were analyzed for CD62L expression after allo-HCT.

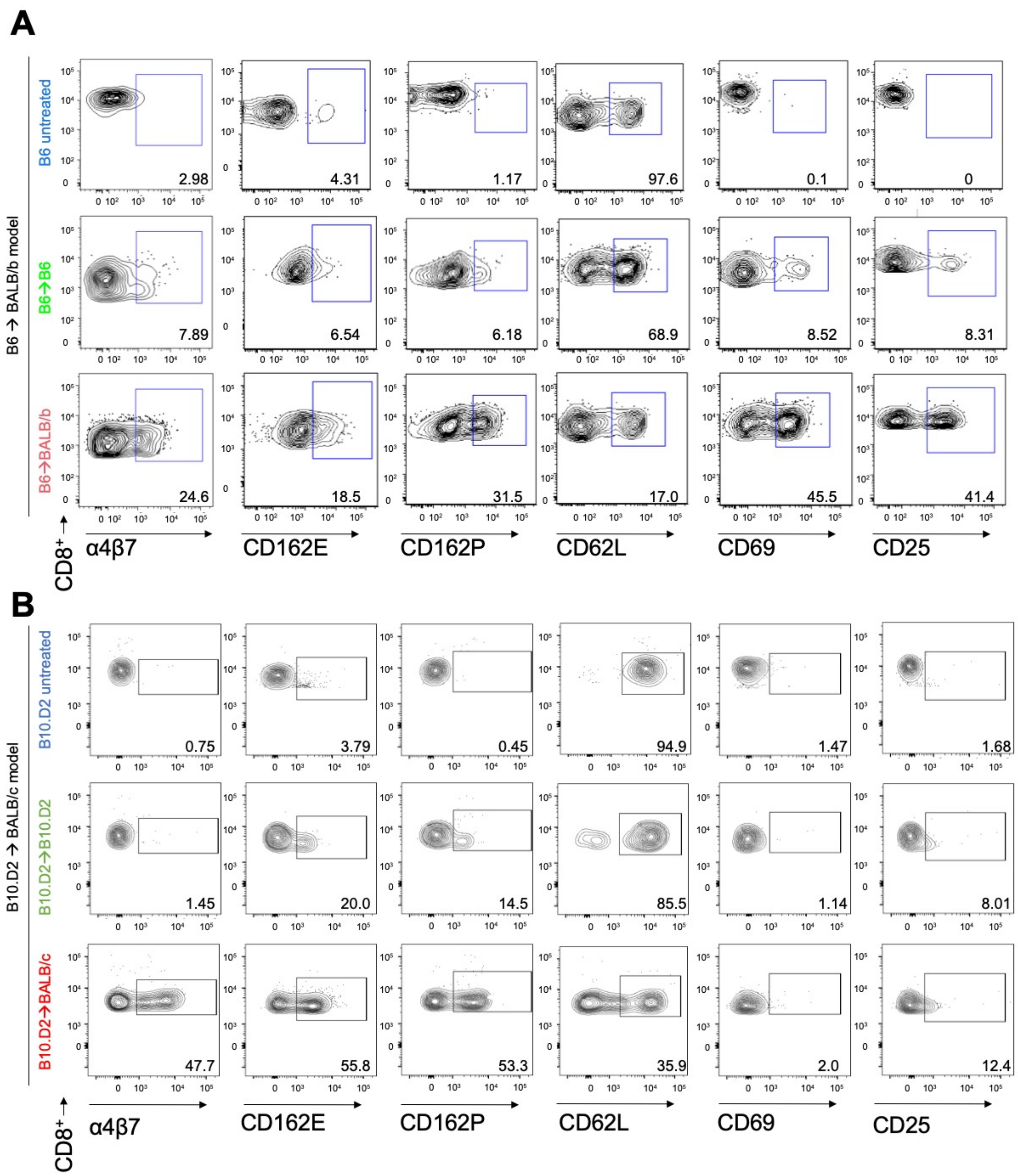


**Supplementary Figure 2: Representative flow cytometry results of peripheral blood CD8$^+$ T cells.**

Surface marker expression of α4β7 integrin, CD162E, CD162P, CD62L, CD69 and CD25 on peripheral blood CD8$^+$ T cells on day+15 after allo-HCT and respective control groups displayed as flow cytometric contour plots with outliers. (**A**) Surface marker expression in untreated B6 healthy controls (top), and on day+15 after B6→B6 syngeneic HCT (middle) or B6→BALB/b miHAg mismatch allo-HCT (bottom). (**B**) Surface marker expression in untreated B10.D2 healthy controls (top), and on day+15 after B10.D2→ B10.D2 syngeneic HCT (middle) or B10.D2→BALB/c miHAg mismatch allo-HCT (bottom).

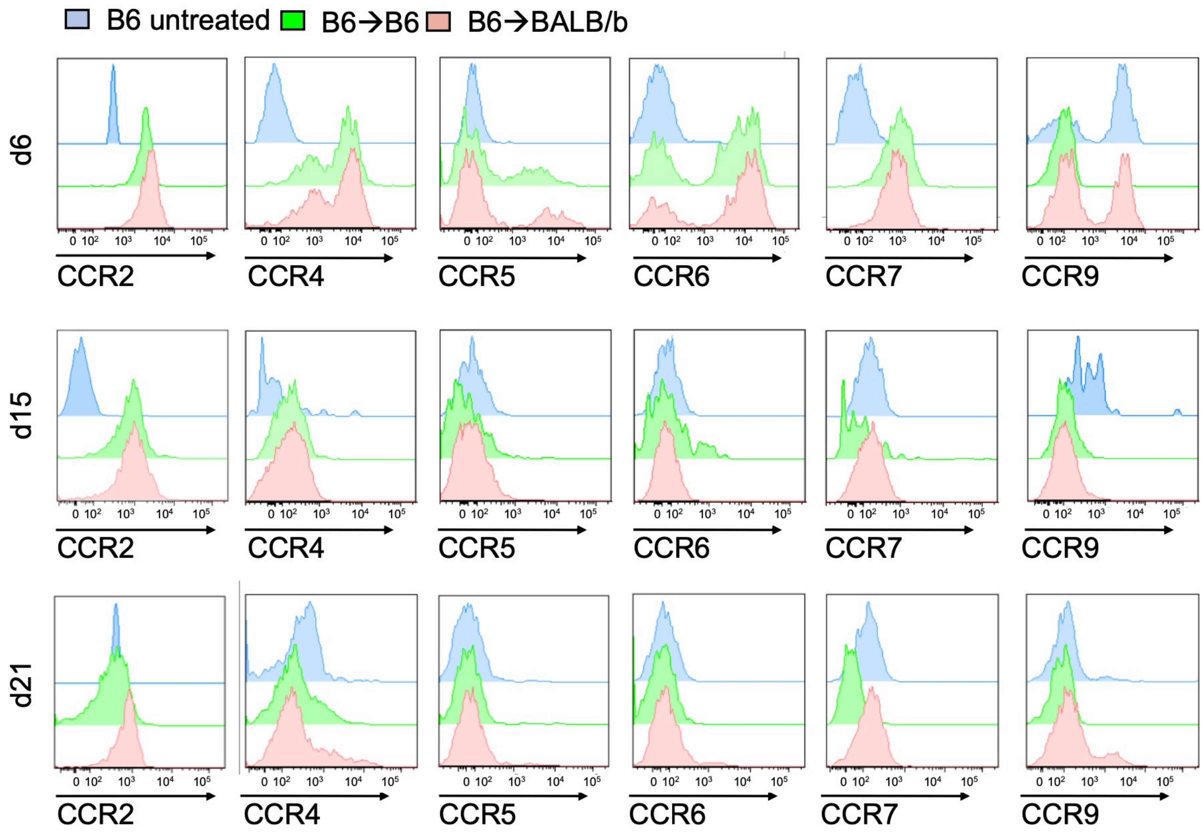


**Supplementary Figure 3: CC-Chemokine receptor expression profiles of donor T-cells do not characterize alloreactive CD8$^+$ T-cells in the peripheral blood.**

Chemokine receptor expression levels on PB CD8$^+$ donor T-cells of B6→BALB/b miHAg mismatch and B6→B6 syngeneic recipients are shown in representative histograms (day +6, +15, +21 after HCT in comparison to untreated B6 controls) (n = 3-10).
